# An oxygen doped porous hard carbon derived from durian shell for high-performance sodium ion storage

**DOI:** 10.1039/d5ra09761d

**Published:** 2026-02-16

**Authors:** Jianliang Guo, Zhihua Sun, Wenzheng Zhu, Lei Li, Song Han, Hongxun Yang

**Affiliations:** a National Energy Group Science and Technology Research Institute Co. Ltd Nanjing Jiangsu 210008 China 12068942@ceic.com; b School of Environmental & Chemical Engineering, Jiangsu University of Science and Technology Zhenjiang Jiangsu 212003 China yhongxun@126.com

## Abstract

Hard carbon is one of the most promising anodes for sodium ion batteries (SIBs) because of its low charge/discharge voltage platform, high specific surface area and higher layer spacing. However, the disadvantages of its unsatisfactory sodium storage capacity and high cost owing to low carbonization yield of precursors frustrate its practical applications. In this paper, we have developed a porous hard carbon derived from durian shell (DSHC) synthesized *via* acid washing and secondary calcination by adjusting carbonization temperature. As an anode for SIBs, the durian shell carbonization at 700 °C (DSHC700) with suitable graphite layer spacing (0.382 nm) delivered a high capacity of 297.2 mAh g^−1^ after 100 cycles at 25 mA g^−1^. The excellent electrochemical performance is attributed to the moderate interlayer spacing due to oxygen doping, and the natural rich porous structure which can increase the active site of Na^+^, shortened the diffusion distance of Na^+^, and promoted the transport of electrolyte. This study could provide a simple method to prepare high performance anodes for SIBs using durian shell as carbon source, and this design strategy could be extended to other biomass-based carbon materials.

## Abstract 2

The optimal as‑obtained durian shell‑derived hard carbon at carbonization temperature of 700 °C delivered a reversible capacity of 297.2 mAh g^−1^ after 100 cycles at a current density of 25 mA g^−1^, demonstrating excellent sodium storage performance.

## Graphical abstract

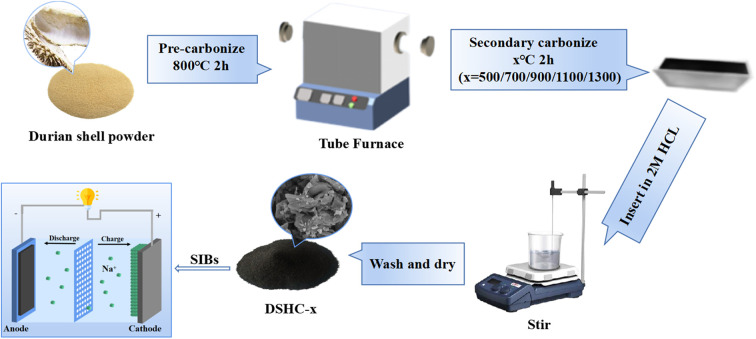

## 1 Introduction

With the rapid development of modern society, people's demand for energy is increasing,^1,2^ and obtaining sustainable new energy has become an important research topic for researchers, such as wind energy, hydropower, solar energy, geothermal energy, tidal energy, *etc.*^3,4^ However, these energy sources have issues such as uncertainty, intermittency, and unsustainability, which greatly limit their widespread application in specific fields.^5–7^ Lithium ion batteries (LIBs) have become the most mature energy storage systems because of their high energy density and long cycle life.^8,9^ They have been widely used in portable electronic devices, large power grids, and other fields. However, due to the limited and uneven distribution of lithium, the high-cost LIBs are unable to meet the growing demand of people.^10,11^ Compared with lithium, sodium has attracted much attention due to its significant advantages such as uniform distribution, and low cost, and is considered an ideal candidate for LIBs.^12–14^ In addition, due to the similar working principle between sodium ion batteries (SIBs) and LIBs, SIBs have become the most promising alternative to LIBs in large-scale energy storage demand.^15,16^ However, the radius of Na^+^(0.103 nm) is larger than that of Li^+^(0.076 nm), while the interlayer spacing of graphite is smaller (0.335 nm),^17–19^ which is not conducive to the insertion and extraction of Na^+^ in the graphite layer when used as a negative electrode material for SIBs.^20,21^ Only a small amount of Na^+^ can be stored in the graphite layer, resulting in the inhibition of reversible capacity. Hard carbon with larger interlayer spacing is an ideal negative electrode material for SIBs and has been widely studied, but its high cost limits its industrial application.^22–24^ Therefore, the development of low-cost SIB negative electrode materials has become a key solution to the current commercialization problem.

Durian is a species of the durian genus, cultivated in southeast Asia, and has been introduced to various parts of the world.^25–27^ In addition to being used as an edible fruit, it is also used in cooking dishes, jams, jellies, cakes, potato chips, sauces, and can be used as an ingredient in ice cream, candies, milkshakes, desserts, and beverages.^28–30^ However, its large-scale application in the food processing industry is hindered by a large amount of durian residues, which mainly exist in the form of durian shells, accounting for 60–75% of the entire durian.^30,31^ In formal practice, a portion of these residues are used as boiler fuel, with the majority being discarded by open-air combustion.^32,33^ Durian shells are composed of 60.5% cellulose, 13.1% hemicellulose, and 15.4% lignin.^25,26^ The high cellulose content ensures a high carbon content, making durian shells as an ideal raw material for producing porous activated carbon.

Herein, we will report a porous hard carbon derived from durian shell (DSHC) through acid washing and calcination by adjusting carbonization temperature. The effect of carbonization temperature on the structure of durian shell based carbon materials was investigated, and their sodium storage mechanism was analyzed in SIBs. When used as the negative electrode for SIBs, the durian shell carbonization at 700 °C (DSHC700) with a suitable graphite layer spacing (0.382 nm) delivered a high capacity of 297.2 mAh g^−1^ after 100 cycles at 25 mA g^−1^. The excellent electrochemical performance is attributed to the moderate interlayer spacing, and the natural rich porous structure which can increase the active site of Na^+^, shortened the diffusion distance of Na^+^, and promoted the transport of electrolyte. This study could provide a simple method to prepare high performance anode materials for sodium ion batteries using biomass durian shell as carbon source, and this design strategy could be extended to other biomass-based carbon materials.

## 2 Experimental section

### 2.1 Materials

Durian shell was purchased from Zhenjiang Fruit Market. Hydrochloric acid (Shanghai Aladdin Biochemical Technology Co., Ltd)was purchased from Shanghai Aladdin Biochemical Technology Co. Ltd.

### 2.2 Synthesis of DSHC

Durian shells were thoroughly clean and then dried in a blast oven at 80 °C for overnight, crushed and passed *via* a 80 mesh screen. Then, 10 g of durian shell powder was annealed in a 800 °C tube furnace for 2 h in argon atmosphere to get black powder. Then, the black powder were soaked in a solution of 2 M HCl and stirred continuously for 6 h, and separated by centrifugation by mixing with deionized water (DI) until the filtrate become to neutral, and then kept in a blast oven at 80 °C for 12 h to obtain the black precursor carbon.

The black precursor carbon was further heated to 500 °C, 700 °C, 900 °C, 1100 °C and 1300 °C to establish a stable carbon framework and regulate the microstructure at a rate of 5 °C min^−1^ in argon atmosphere for 2 h, respectively, and then cooled naturally to room temperature to obtain hard carbon derived from durian shells. The samples carbonized at different temperatures were labeled as DSHC-*x* (*x* is calcination temperature, 500 °C, 700 °C, 900 °C, 1100 °C and 1300 °C).

### 2.3 Materials characterization

Using a field emission scanning electron microscope (FESEM), the surface particle size and distribution of the material are precisely observed. Further analysis of the material's internal structure is conducted using the Hitachi HT7800 Transmission Electron Microscope (TEM). The crystalline structure of the material is analyzed by scanning in the 2*θ* range of 10° to 80° using the Shimadzu PXRD-6000 X-ray powder diffractometer (scanning rate: 8° min^−1^). Molecular structure analysis is carried out using the Renishaw1000 Raman spectrometer, and the surface area and pore size distribution are measured using the Quantachrome Instruments V-sorb 800 surface area and pore size analyzer. The elemental composition and chemical states of the material are deeply analyzed using the Shimadzu AXIS X-ray photoelectron spectrometer (XPS).

### 2.4 Electrochemical measurements

The sodium storage performances of the DSHC-*x* were evaluated by assembling CR2032 button half-cell with sodium sheet as counter electrode. In order to prepare the working electrode, 80 wt% active material, 10 wt% acetylene black and 10 wt% PVDF were ground and mixed, and *N*-methylpyrrolidone was added for continuous stirring for 12 hours to obtain a slurry containing active material. Then the slurry was coated on the copper foil according to a certain thickness, dried in the shade and vacuum dried at 80 °C for 12 hours. The unit mass density of the material on the copper foil is about 1.1 mg cm^−2^. The electrolyte of sodium ion battery is EC/PC mixed solvent containing 1 M NaClO_4_ (volume ratio of 1 : 1).

## 3 Results and discussion

### 3.1 Structural characterizations and analyses

**Scheme 1:**
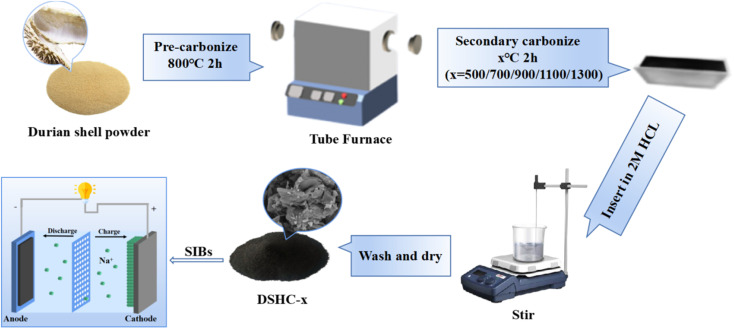
Preparation process of durian shell-based hard carbon and schematic diagram of Na//DSHC half-cell.

**Fig. 1:**
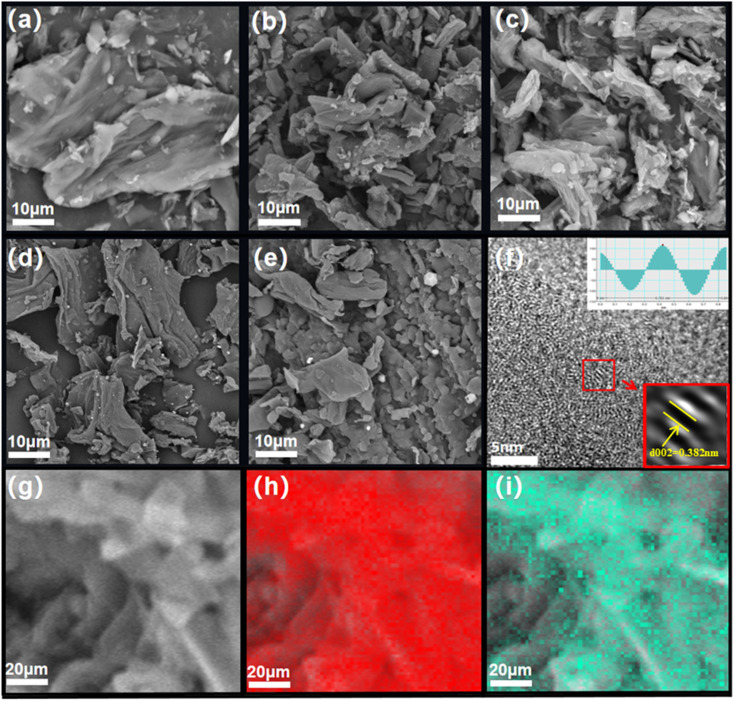
(a–e) SEM pictures of DSHC500, DSHC700, DSHC900, DSHC1100 and DSHC1300; (f) HRTEM images of DSHC700; (g) element mapping selected area; (h and i) red and green represent C and O, respectively.

Scheme 1 shows the synthesis process of DSHC-*x* and its application in SIBs. Fig. 1a exhibits the micromorphologies of DSHC-*x*. From Fig. 1a–e, it can be observed that the SEM images of the DSHC-*x* samples composed of irregular flake-like structures. With the increase of the secondary calcination temperature, the surface morphology of the DSHC-*x* samples tends towards more ordered structures, resulting in a tighter connection between the flake-like structures. The block-like structures are most prominent at 500 °C, possibly due to insufficient calcination temperature. We further conducted a thorough analysis of the well-performing DSHC700 from the perspectives of microstructure, composition, and defects using high-resolution transmission electron microscopy (HRTEM). From Fig. 1f, it can be observed that the DSHC700 is enveloped by densely packed pore structures, which are somewhat dispersed in certain areas while densely distributed in others. Therefore, overall observation of this pore distribution does not follow a clear pattern. Upon further observation of the gap between carbon layers, a larger lattice spacing of 0.382 nm is computed by the computer, being attributed to the doping of oxygen heteroatoms.^4,34,35^ For Na^+^ ions with relatively large atomic radii, such wide pores are more conducive to the extraction/insertion of Na^+^ ions, significantly improving and enhancing the diffusion efficiency of ions. Additionally, such uniform pore distribution not only can accommodate the volume expansion caused by the charging/discharging process of hard carbon, but also provide better transmission space for the electrolyte, accelerating charge transfer rates and enhancing transmission stability.^36^Fig. 1g–i depict the elemental mapping regions and corresponding elemental distribution of the DSHC700. EDS characterization clearly reveals the homogeneous distribution of oxygen and carbon elements on the DSHC700 material. The oxygen in DSHC700 mainly comes from the natural oxygen-containing functional groups (such as hydroxyl OH, carboxyl COOH, carbonyl–C=O, *etc.*) of its precursors (lignin, cellulose). During the pyrolysis carbonization process, these functional groups are not completely decomposed, but exist in the form of residual oxygen in the hard carbon structure. Actually, the oxygen in biomass hard carbon significantly can enhances the specific capacity, rate performance, and interfacial stability of SIBs by regulating the microstructure (interlayer spacing, defects) and surface chemistry (functional groups, wettability), which is one of the key advantages that distinguish natural biomass carbon materials from graphite or synthetic hard carbon.^35^ The electrochemical performances can be further optimize by precisely regulating the form and content of oxygen, such as calcination temperature.

**Fig. 2:**
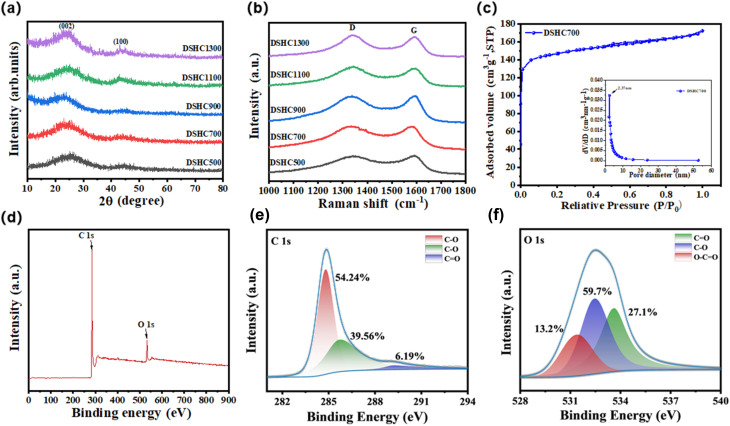
(a) XRD patterns of DSHC-*x* composites; (b) Raman spectra of DSHC-*x* composites. (c) N_2_ adsorption–desorption isotherms and pore size distribution of DSHC700 composites; (d) XPS full spectrum of DSHC700; (e) C 1s high resolution spectra of DSHC700; (f) O 1s high resolution spectra of DSHC700.

To deeply explore the internal microstructures of these DSHC-*x* composites, we conducted XRD spectra, as shown in Fig. 2a. It is obviously seen that all five DSHC-*x* behave distinct peaks near 24° and 44°. The peak around at 24° corresponds to the (002) characteristic peak, representing the (002) crystal plane diffraction peak of hard carbon.^37^ This peak reflects the distribution of the internal lattice and layered structure, while the XRD spectrum at 44° corresponding to the (100) characteristic peak more clearly indicates the presence of graphite crystal structures within the hard carbon.^38,39^ We extracted the (002) peak value and, using Bragg's law equation (Text S1), calculated the *d*_002_ values for DSHC500, DSHC700, DSHC900, DSHC1100, and DSHC1300 to be 0.378 nm, 0.381 nm, 0.391 nm, 0.367 nm, and 0.362 nm, respectively (Table S1). It is noted that when the material synthesis temperature exceeds 1000 °C, the smaller the interlayer spacing, indicating that the high temperature leads to a collapse of carbon layers, resulting in smaller lattice spacing. In comparison, the samples calcined at 500 °C, 700 °C, and 900 °C showed more larger lattice spacing. We also conducted Raman spectroscopy analysis on these DSHC-*x* composite. As shown in Fig. 2b, the Raman spectra of the five samples at different secondary calcination temperature exhibit a similar trend, with two distinct diffraction peaks observed around 1350 cm^−1^ and approximately 1590 cm^−1^, which represent the typical D band and G band of hard carbon, respectively.^4,40^ The D band indicates defects and some amorphous phases in the hard carbon materials, with a higher intensity of the D peak suggesting a higher defect density. Meanwhile, the vibration of sp^2^ hybridized carbon atoms inside the hard carbon materials causes the G band, usually reflecting the degree of graphitization and the characteristics of the crystal structure. By using *I*_D_/*I*_G_ ratio, we can specifically determine the composition of the materials, with *I*_D_/*I*_G_ values for DSHC500, DSHC700, DSHC900, DSHC1100, and DSHC1300 calculated as 1.03, 1.01, 1.00, 1.11, and 1.08, respectively (Table S1). These results indicate that the DSHC-*x* composites possess disordered structural characteristics, indicating a more disordered structural feature. We further calculated the crystallite size (*L*_a_, nm) using the *I*_D_/*I*_G_ values, an important parameter describing the characteristics of the crystal structure, reflecting the reference value of the material's crystal particle size and average size.^16^ According to the formula in Text S2, the *L*_a_ values for DSHC500, DSHC700, DSHC900, DSHC1100, and DSHC1300 are 19.697 nm, 18.978 nm, 19.167 nm, 17.319 nm, and 17.801 nm, respectively.^40^ Comparatively, DSHC1100 and DSHC1300 have smaller *L*_a_ values, indicating that the growth of these materials is restricted and their crystal structures are relatively incomplete. In contrast, the higher *L*_a_ values of DSHC500 and DSHC900 imply a more ordered structure, while the lower *L*_a_ value of DSHC700 suggests a more disordered internal structure with a good content of sp^2^ hybridized carbon atoms, which can provide more free space for ions, accelerating electrolyte transport, consistent with previous analyses.

Further exploration using BET and BJH analyses on the DSHC700 revealed a high surface area of 512.25 m^2^ g^−1^ and a rich pore structure as shown in Fig. 2c. The high BET can provide more space for Na^+^ intercalation/deintercalation, allow the electrode material to be fully absorbed and contacted by the electrolyte,^41^ and enhance ion transport efficiency, and thereby improving cyclic performance. This porosity also helps to provide Na^+^ ions with more room to move, allowing for shorter ion transport and diffusion paths compared to microstructures with fewer pores. The DSHC700 not only contains a small amount of micropores but also many large mesopores, mostly with diameters between 2–30 nm, due to the gaps between carbon particles, and the presence of numerous mesopores can provide more active sites for Na^+^. Fig. 2d shows the full spectrum XPS diagram of the DSHC700 to investigate the chemical composition and state. It can be clearly seen that there are two distinct peaks through fitting analysis as C 1s and O 1s peaks, located at 285.08 eV and 533.08 eV, respectively. This confirms that the DSHC700material is composed of carbon and oxygen, consistent with previous analyses. The high-resolution C 1s spectrum of DSHC700 in Fig. 2e includes three bonds: C–C (∼284.8 eV), representing the content in the defect region, C–O (∼285.78 eV), and C=O (∼290 eV). The presence of C–C provides support for the material's overall structure and maintains the chemical stability within, while during the charge–discharge cycle of the battery.^42,43^ The C–O reacts with Na^+^ to promote ion activity and affect the internal charge distribution and electron transfer, and C=O is involved in more complex redox reactions to enhance electrochemical performance.^44–46^Fig. 2f shows the presence of oxygen, helping to expand interlayer spacing to promote Na^+^ embedding, provide surface active sites to enhance pseudocapacitive contribution, improve electrolyte wettability, and inhibit side reaction. The DSHC700 maintains a moderate oxygen content with a balanced proportion of electrochemically active oxygen species, which contributes to an appropriate interlayer spacing and abundant Na^+^ adsorption sites, leading to superior electrochemical performance.

### 3.2 Electrochemical performances

**Fig. 3:**
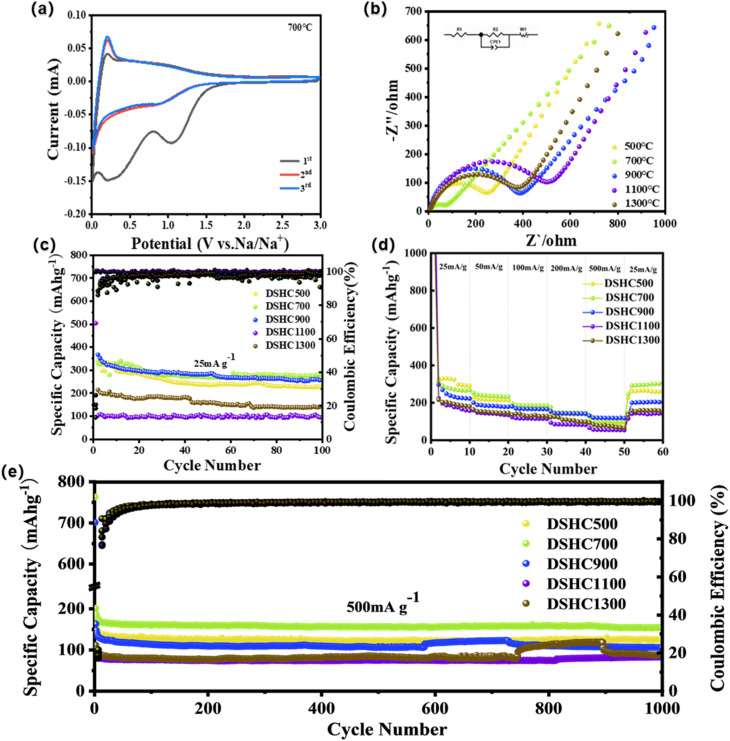
(a) The first 3rd CV curves of the DSHC700 at a scanning rate of 0.1 mVs^−1^; (b) the EIS spectra of DSHC-*x* before cycling; (c) the cycle performances of DSHC-*x* at a current density of 25 mA g^−1^; (d) the rate performances of DSHC-*x* at the different current densities of 25 mA g^−1^,50 mA g^−1^,100 mA g^−1^,200 mA g^−1^, and 500 mA g^−1^, respectively; (e) the long cycle performances of DSHC-*x* at a current density of 500 mA g^−1^.

Fig. 3a presents the cyclic voltammetry (CV) curves of DSHC700 tested at a voltage range of 0.01–3 V and a scan rate of 0.1 mV, displaying the typical CV characteristics of hard carbon materials. The similarity in the CV curves across the five DSHC-*x* samples indicates their analogous electrochemical properties (Fig. S1). In the first cycle, two reduction peaks were observed in all electrodes between 1.0–1.5 V, being attributed to the interaction of Na^+^ insertion with oxygen-containing groups, while a reduction peak appeared within the 0.2–0.5 V range due to the formation of the solid–electrolyte interface (SEI) layer.^16,44^ The absence of reduction peaks in the 1.0–1.5 V range during the first discharge suggests the formation of a more stable and irreversible SEI layer, with the peak at 0.01 V indicating the gradual insertion of Na^+^ into the carbon to form NaC_*x*_ compounds, and an oxidation peak around 0.15 V corresponding to Na^+^ extraction from hard carbon. Subsequent CV cycles almost perfectly overlap, indicating good kinetic performance and cycling stability of the electrodes.

For the sodium-ion half-cell assembled with DSHC700, we analyzed the voltage–capacity relationship across the 1st, 2nd, 50th, and 100th cycles tested at a current density of 25 mA g^−1^, as depicted in Fig. S2a. The DSHC700 exhibited a high discharge capacity of 1689.4 mAh g^−1^ in the first cycle due to the presence of porosity and a high surface area. While the first charge cycle capacity was only 312.5 mAh g^−1^ with a low initial coulombic efficiency (ICE) of 18.5%. It should be noted that the ICE is lower than some reported carbon materials (Table S2).^43–53^ The low ICE mainly stems from three core factors: the high specific surface area and hierarchical porous structure of biomass carbon expand the electrode–electrolyte contact area, exacerbating excessive SEI formation on pore surfaces; in addition, incomplete carbonization and tiny micropores in the carbon matrix lead to irreversible Na^+^ trapping and residual organic decomposition, causing further irreversible capacity loss; oxygen-containing functional groups in biomass carbon trigger irreversible side reactions with electrolytes during the first Na^+^ intercalation, consuming active Na^+^ for the formation of solid electrolyte interphase (SEI) films.^44^ The DSHC700 sample behaved a discharging voltage plateau of 1.75 V and charging voltage plateau of 0.25 V, suggesting irreversible capacity loss, which aligns with the CV curve results. The discharge capacity normalized to 330 mAh g^−1^ in the second cycle and gradually improved, stabilizing above 98.5%, indicating gradual formation of the SEI layer on the electrode surface. The near-identical charge–discharge curves at the 50th and 100th cycles demonstrate the structural stability of the as-prepared hard carbon material, further proving the excellent reversibility of the DSHC700. Meanwhile, Meanwhile, the voltage-capacity curves at the 1st, 2nd, 50th, 100th, 500th and 1000th cycle are provided (Fig. S2). It can be clearly observed that the charge curves from 2nd cycle almost overlap, indicating that the DSHC-*x* materials exhibit good capacity retentions and overall electrochemical performances.

The conductivity of DSHC-*x* electrode was studied through electrochemical impedance spectroscopy (EIS) testing. As shown in Fig. 3b and S3, the curves plotted for the five DSHC-*x* samples consist of a semicircle and a diagonal line, corresponding to the resistance in the high and low frequency regions, respectively.^48^ From the graph, it can be roughly seen the magnitude of the charge transfer resistance (*R*_ct_) of the material and the diffusion impedance (*R*_s_). As shown in Table S3, the *R*_ct_ values of the DSHC700 is much smaller than other four DSHC-*x* samples at 25 mA g^−1^ after 100 cycles. As the number of cycles increased, the electrolyte continued to diffuse to the interior, promoting the gradual activation of the material and improving the electron transfer efficiency inside the battery. The lower the *R*_ct_ and *R*_s_ values of the DSHC700, the better its conductivity. In this regard, due to its internal structure being more conducive to the transport of sodium ions, the *R*_ct_ and *R*_s_ values of DSHC700 after cycling are significantly lower than other materials, thus exhibiting better electrochemical performance.

In order to investigate the effect of temperature on the sodium storage capacity of DSHC-*x* series electrodes, cyclic tests were conducted on DSHC-*x* at a current density of 25 mA g^−1^, and the results are shown in Fig. 3c. The initial charge/discharge capacities of DSHC500, DSHC700, DSHC900, DSHC1100, and DSHC1300 are 209.9/1602.2 mAh g^−1^, 312.5/1689.4 mAh g^−1^, 361.3/1747 mAh g^−1^, 179.9/1044.7 mAh g^−1^, and 192.5/732.3 mAh g^−1^, with initial coulombic efficiencies of 13.1%, 18.5%, 20.68%, 17.2%, and 26.29%, respectively. It has been observed that the DSHC-*x* series materials exhibit lower initial coulombic efficiency, mainly being due to the formation of SEI film and electrolyte decomposition during battery cycling. In subsequent cycling, the coulombic efficiency gradually increases due to structural stability. It should be noted that the specific capacity of the DSHC700 sample can still maintain 280.2 mAh g^−1^ even after 100 cycles, which is higher than other DSHC-*x* samples and some previous carbon materials derived-from different biomass sources (Table S2). This result shows the outstanding excellent lithium storage capability and cycling performances of DSHC700.

We chose the rate performances of electrodes as the research object and tested the cycling capacity of DSHC500, DSHC700, DSHC900, DSHC1100, and DSHC1300 at the various current densities. We aim to delve deeper into the differences in performance between them. According to the data in Fig. 3d, the specific capacity of DSHC700 slightly decreased in the first cycle at a current density of 25 mA g^−1^, with a decrease of nearly 11.8% from the initial discharge capacity of 383.5 mAh g^−1^. As the cycle progressed, the SEI film formed by the electrode material and some side reactions gradually occurred, leading to a change in capacity. At a current density of 50 mA g^−1^, the capacity of the DSHC700 electrode is maintained at 306.4 mAh g^−1^. As the current density increases to 100 mA g^−1^ and 200 mA g^−1^, its cycling capacity decreases to 255.2 mAh g^−1^ and 216.8 mAh g^−1^, respectively. When the current density reaches 500 mA g^−1^, the capacity of the DSHC700 electrode further decreases to 170.2 mAh g^−1^. Finally, when the current density returned to 25 mA g^−1^, its cycling capacity returned to 361.9 mAh g^−1^, which is basically consistent with the cycling performance of the previous 25 mA g^−1^, indicating that the DSHC700 has excellent cycling rate performance. Fig. 3e shows the cycling performance of DSHC500, DSHC700, DSHC900, DSHC1100, and DSHC1300 electrodes at a high rate of 500 mA g^−1^. After 1000 cycles, the specific capacities of the DSHC500, DSHC700, DSHC900, DSHC1100, and DSHC1300 electrodes remained and stabilized at 124.8 mAh g^−1^, 153.2 mAh g^−1^, 105.6 mAh g^−1^, 82.6 mAh g^−1^, and 88.1 mAh g^−1^, respectively. In contrast, the DSHC700 can still maintain excellent cycling performance at high current densities, indicating that DSHC700 not only exhibits good performance at low currents, but also can cope with capacity storage at high current densities. The superior sodium storage performance of DSHC700 is ascribed to the result of a synergistic balance among the pore structure, interlayer spacing, oxygen doping, and structural integrity.

**Fig. 4:**
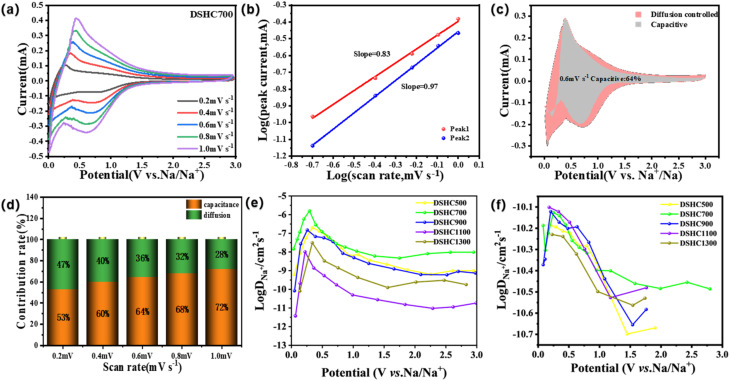
(a) Cyclic voltammetry curves of the DSHC700 electrode at scanning rates of 0.1, 0.2, 0.4, 0.6, 0.8 and 1.0 mV s^−1^; (b) logarithmic relationship between scanning rate and peak current of DSHC700 electrode and linear fitting of b value; (c) the contribution of capacitance behavior in the cyclic voltammetry curve of DSHC700 electrode at 0.8 mV s^−1^; (d) the capacitance contribution of DSHC700 electrode at 0.1–1.0 mV s^−1^; (e) apparent diffusion coefficient of Na^+^ in DSHC-*x* during charging; (f) apparent diffusion coefficient of Na^+^ in DSHC-*x* during discharge.

To gain deeper insights into the electrode kinetics, we conducted tests on the DSHC700 electrode within the voltage range of 0.01 to 3 V, employing a scanning rate of 0.2 mV s^−1^ by 0.2 mV increments until reaching 1.0 mV s^−1^ for CV measurements. As depicted in Fig. 4a, the area under the CV closed curves continuously increased with increasing scan rate, indicating a correlation between scan rate and the electrode's electrochemical reaction capability due to the influence of diffusion and electron transfer processes at higher scan rates, resulting in decreased electrochemical reaction ability for the battery. Typically, the total electrode capacitance comprises two parts: the ion adsorption-controlled capacitance and the ion diffusion-controlled capacitance. The relationship between peak current (*i*) and scan rate (*v*) can be analyzed in detail using Text S3, where a value close to 0.5 suggests diffusion-controlled processes dominating the electrochemical reaction, while a value close to 1 indicates the predominance of capacitive processes.^23,48^ By analyzing the CV curves, the relationship between the logarithm of current density (log(*i*)) and the logarithm of scan rate (log(*v*)) can be observed to determine the control mechanism of the electrochemical reaction. As shown in Fig. 4b, the capacitive contribution of the DSHC700 electrode exhibits ion adsorption-controlled capacitance behavior combined with ion diffusion control. Using Text S4, we can accurately calculate the contribution rates of electrode capacity-related components, enabling the estimation of currents *k*_1_ and *k*_2_ to determine pseudocapacitive behavior and the resulting current.^49,50^Fig. 4c illustrates the relationship between the pseudocapacitive contribution area (gray area) and the diffusion contribution area (pink area) at a scan rate of 0.6 mV s^−1^, with the pseudocapacitive contribution area reaching 64%, indicating its dominant behavior. According to Fig. 4d, as the scan rate increases, the contribution rate of pseudocapacitive behavior continually increases, indicating that the surface control effect increasingly determines the size of the capacity contribution as the scan rate increases. The DSHC700 exhibits excellent rate performance and pseudocapacitive behavior at different scan rates, demonstrating good dynamic performance.

Further exploration of the dynamic characteristics of DSHC700 was conducted using the galvanostatic intermittent titration technique (GITT), which studies the voltage variation of SIBs at constant current at each interval to analyze the diffusion coefficient of Na^+^ (D_Na^+^_) (Fig. S4). D_Na^+^_ was calculated using Fick's second law (Text S5), Δ*E*_s_ and Δ*E*_*τ*_ were obtained from the voltage variation within single time intervals from the GITT curve. The GITT curve reflects the trend of voltage variation in the material over a period, where DSHC700 exhibited significantly longer recording times and smaller voltage variations compared to the other four samples. This result indicated a larger amount of Na^+^ storage and higher ion output capacity of DSHC700, whereas DSHC1100 and DSHC1300 performed the worst. The relatively stable open circuit voltage also confirms the good electrochemical stability of DSHC700. Plotting the relationship between D_Na^+^_ and voltage (Fig. 4f and g), the diffusion coefficient of DSHC700 is significantly higher than that of the other samples, indicating its excellent diffusion performance. The outstanding dynamic performance of DSHC700 may be attributed to an appropriate number of micropores and mesopores, which provide effective channels for ion recombination and facilitate electrolyte penetration. Therefore, the DSHC700 exhibits significant potential as a negative electrode material for SIBs.

## 4 Conclusions

In summary, we utilized durian shell as a carbon source to prepare a series of durian shell-based negative electrode for SIBs *via* acid washing and adjusting the carbonization temperature for secondary annealing. The effect of carbonization temperature on the structure of durian shell-based carbon materials was investigated, and its sodium storage mechanism in SIBs as well as its impact on the electrochemical properties of the material were analyzed. Employed as the negative electrode for SIBs, the DSHC700 delivered a reversible capacity of 297.2 mAh g^−1^ after 100 cycles at a current density of 25 mA g^−1^. Even after 1000 cycles at 500 mA g^−1^, its capacity could still reach 163.7 mAh g^−1^. The excellent electrochemical performance is attributed to moderate interlayer spacing (0.382 nm), and the natural rich porous structure which can increase the active site of Na^+^, shortened the diffusion distance of Na^+^, and promoted the transport of electrolyte. This work provides a possible approach for designing environmentally friendly, low-cost, high-performance negative electrode for SIBs, which is of significant importance for the future application of carbon-based materials in sodium ion energy storage.

## Conflicts of interest

The authors claim that they have no known financial or interpersonal conflicts that might have an impact on the research presented in this publication.

## Supplementary Material

RA-016-D5RA09761D-s001

## Data Availability

The data supporting this article have been included as part of the supplementary information (SI). Supplementary information is available. See DOI: https://doi.org/10.1039/d5ra09761d.

## References

[cit1] Zhou S., Zhou L., Zhang Y., Sun J., Wen J., Yuan Y. (2019). Upgrading earth-abundant biomass into three-dimensional carbon materials for energy and environmental applications. J. Mater. Chem. A.

[cit2] Tian H., Sun Z., Ren L., Jin Y., Wang D., Wei Y., Chen H., Liu K., Chen Y., Yang H. (2024). Hollow CoSe_2_-ZnSe microspheres inserted in reduced graphene oxide serving as advanced anodes for sodium ion batteries. J. Colloid Interface Sci..

[cit3] Li L., Sun M., Xu Z., Wang Z., Liu K., Chen Y., Wang Z., Chen H., Yang H. X. (2023). Hierarchical porous hard carbon derived from rice husks for high-performance sodium ion storage. Colloids Surf., A.

[cit4] Zou X. F., Dong C., Jin Y. C., Wang D., Li L., Wu S. T., Xu Z. Z., Chen Y. Y., Li Z. H., Yang H. X. (2023). Engineering of N, P co-doped hierarchical porous carbon from sugarcane bagasse for high-performance supercapacitors and Sodium-ion batteries. Colloids Surf., A.

[cit5] Li Y., Chen M., Liu B., Zhang Y., Liang X., Xia X. (2020). Heteroatom Doping: An Effective Way to Boost Sodium Ion Storage. Adv. Energy Mater..

[cit6] Luo L., Lan Y., Zhang Q., Deng J., Luo L., Zeng Q., Gao H., Zhao W. (2022). A review on biomass-derived activated carbon as electrode materials for energy storage supercapacitors. J. Energy Storage.

[cit7] Jin Y. C., Wu S. T., Wang Y. Y., Xu Z. Z., Chen L. Z., Yang H. X. (2024). N/P/O co-doped porous carbon derived from agroindustry waste of peanut shell for Sodium-ion storage. J. Energy Storage.

[cit8] Wang Y., Guo X., Wang Z., Lü M., Wu B., Wang Y., Yan C., Yuan A., Yang H. (2017). Controlled pyrolysis of MIL-88A to Fe_2_O_3_@C nanocomposites with varied morphologies and phases for advanced lithium storage. J. Mater. Chem. A.

[cit9] Sun M., Xu Z., Liu K., Yang H., Yang T., Jin C., Wang Z., Jin Y., Chen L. (2022). Construction of rice husk-derived SiOx nanoparticles encapsulated with graphene aerogel hybrid for high-performance lithium ion batteries. Electrochi. Acta.

[cit10] Wang Y., Zhu W., Wang G., Chen C., Xu W., Li Y., Chen L., Chen Y., Yang H. (2025). A new N/O Co-doped soft-hard carbon nanofibers composite for advanced sodium storage. J. Energy Storage.

[cit11] Zheng Y., Chen K., Jiang K., Zhang F., Zhu G., Xu H. (2022). Progress of synthetic strategies and properties of heteroatoms-doped (N, P, S, O) carbon materials for supercapacitors. J. Energy Storage.

[cit12] Yeletsky P. M., Lebedeva M. V., Yakovlev V. A. (2022). Today's progress in the synthesis of porous carbons from biomass and their application for organic electrolyte and ionic liquid based supercapacitors. J. Energy Storage.

[cit13] Tian H., Bao P., Li Y., Chen Y., Sun D., Yang H. (2025). Nitrogen-doped carbon nanofibers defining heterogeneous Fe_3_Se_4_-NiSe_2_ nanoparticles as anodes for sodium ion batteries. J. Energy Storage.

[cit14] Huang Q., Liu G., Xie Z. (2022). Properties
of silicon-based lithium batteries with different electrode nanostructures. J. Phys., Conf. Ser..

[cit15] Tian H., Xu Z., Liu K., Wang D., Ren L., Wei Y., Chen L., Chen Y. Y., Liu S., Yang H. (2024). Heterogeneous bimetallic selenides encapsulated within graphene aerogel as advanced anodes for sodium ion batteries. J. Colloid Interface Sci..

[cit16] Li Y. J., Zou X. F., Li S. Q., Chen Y. Y., Wang G. X., Yang H. X., Tian H. (2024). Biomass-derived B/N/P co-doped porous carbons as bifunctional materials for supercapacitors and sodium-ion batteries. J. Mater. Chem. A.

[cit17] Wang X., Wang L., Wan J., Zhou P., Chen J., Gong Y., Xu K. (2019). Corrugated Paper-Based Activated Carbon as a Bifunctional Material for the Electrocatalytic Degradation and High-Performance Supercapacitors. J. Electrochem. Soc..

[cit18] Zhou J., Ye S., Zeng Q., Yang H., Chen J., Guo Z., Jiang H., Rajan K. (2020). Nitrogen and Phosphorus Co-doped Porous Carbon for High-Performance Supercapacitors. Front. Chem..

[cit19] Guo D., Li Z., Liu P., Sun M. (2021). N,P,S co-doped biomass-derived hierarchical porous carbon through simple phosphoric acid-assisted activation for high-performance electrochemical energy storage. Int. J. Hydrogen Energ..

[cit20] Liang X., Liu R., Wu X. (2021). Biomass waste derived functionalized hierarchical porous carbon with high gravimetric and volumetric capacitances for supercapacitors. Microporous Mesoporous Mater..

[cit21] Chen S., Wang S., Peng Q., Wei Z., Cheng S., Fang Z., Duan P., Cheng Y., Cheng Y., Jin K., Jiang L., Wang Q. (2024). In-situ fabricated succinonitrile-based composite electrolyte for high-performance and safe solid-state lithium batteries. J. Energy Storage.

[cit22] Lu Y., Zhu T. (2024). Status and prospects of lithium iron phosphate manufacturing in the lithium battery industry, Mrs. Commun.

[cit23] Li Q., Zhang Y.-N., Feng S., Liu D., Wang G., Tan Q., Jiang S., Yuan J. (2021). N, S self-doped porous carbon with enlarged interlayer distance as anode for high performance sodium ion batteries. Int. J. Energy Res..

[cit24] Jamesh M. I., Prakash A. S. (2018). Advancement of technology towards developing Na-ion batteries. J. Power Sources.

[cit25] Foo K. Y., Hameed B. H. (2012). Textural porosity, surface chemistry and adsorptive properties of durian shell derived activated carbon prepared by microwave assisted NaOH activation. Chem. Eng. J..

[cit26] Tan Y. L., Abdullah A. Z., Hameed B. H. (2017). Fast pyrolysis of durian (Durio zibethinus L) shell in a drop-type fixed bed reactor: Pyrolysis behavior and product analyses. Bioresour. Technol..

[cit27] Xue Y., Gao M., Wu M., Su D., Guo X., Shi J., Duan M., Chen J., Zhang J., Kong Q. (2020). A Promising Hard Carbon-Soft Carbon Composite Anode with Boosting Sodium Storage Performance. ChemElectroChem.

[cit28] Gao X., Zheng Z., Pan Y., Song S., Xu Z. (2025). Aligned Hollow Silicon Nanorods Containing Ionic Liquid Enhanced Solid Polymer Electrolytes with Superior Cycling and Rate Performance. Adv. Sci..

[cit29] Gautam M., Mishra G. K., Furquan M., Bhawana K., Kumar D., Mitra S. (2023). Design of Low-Stress robust silicon and Silicon-Carbide
anode with high areal capacity and high energy density for Next-Generation Lithium-Ion batteries. Chem. Eng. J..

[cit30] Yuan M., Cao B., Meng C., Zuo H., Li A., Ma Z., Chen X., Song H. (2020). Preparation of pitch-based carbon microbeads by a simultaneous spheroidization and stabilization process for lithium-ion batteries. Chem. Eng. J..

[cit31] Luo J., Ma B., Peng J., Wu Z., Luo Z., Wang X. (2019). Modified Chestnut-Like Structure Silicon Carbon Composite as Anode Material for Lithium-Ion Batteries. ACS Sustainable Chem. Eng..

[cit32] Baldan M. R., Almeida E. C., Azevedo A. F., Gonçalves E. S., Rezende M. C., Ferreira N. G. (2007). Raman validity for crystallite size La determination on reticulated vitreous carbon with different graphitization index. Appl. Surf. Sci..

[cit33] He Q., Yu J., Wang Y., Zhong Z., Jiang J., Su F. (2018). Silicon nanoparticles prepared from industrial wastes as high-performing anode materials for lithium ion batteries. Solid State Ionics.

[cit34] Mittal U., Djuandhi L., Sharma N., Andersen H. L. (2022). Structure and function of hard carbon negative electrodes for sodium-ion batteries. J. Phys. Energy.

[cit35] Zhang Y., Zhang N., Chen W., Rao Z., Wu J., Xue L., Zhang W. (2019). Effect of Vapor Carbon Coating on the Surface Structure and Sodium Storage Performance of Hard Carbon Spheres. Energy Technol..

[cit36] Tang X., Xie F., Lu Y., Chen Z., Li X., Li H., Huang X., Chen L., Pan Y., Hu Y. S. (2023). Intrinsic effects of precursor functional groups on the Na storage performance in carbon anodes. Nano Res..

[cit37] Li Z., Qi S., Liang Y., Zhang Z., Li X., Dong H. (2019). Plasma Surface Functionalization of Carbon Nanofibres with Silver, Palladium and Platinum Nanoparticles for Cost-Effective and High-Performance Supercapacitors. Micromachines.

[cit38] Hu W., Chen N., Chen D., Tong B. (2022). Conjugated Tetrathiafulvalene Carboxylates for Stable Organic Lithium Batteries. ChemElectroChem.

[cit39] Rahman M. O., Nor N. B., Sawaran Singh N. S., Sikiru S., Dennis J. O., Shukur M. F. b. A., Junaid M., Abro G. E. M., Siddiqui M. A., Al-Amin M. (2023). One-Step Solvothermal Synthesis by Ethylene Glycol to Produce N-rGO for Supercapacitor Applications. Nanomaterials.

[cit40] Xia K., Huang Z., Zheng L., Han B., Gao Q., Zhou C., Wang H., Wu J. (2017). Facile and controllable synthesis of N/P co-doped graphene for high-performance supercapacitors. J. Power Sources.

[cit41] Lu Z., Liu X., Wang T., Huang X., Dou J., Wu D., Yu J., Wu S., Chen X. (2023). S/N-codoped carbon nanotubes and reduced graphene oxide aerogel based supercapacitors working in a wide temperature range. J. Colloid Interface Sci..

[cit42] Zhou Y., Wang P., Wang K., Fang X., Li W., Nai J., Liu Y., Wang Y., Zou S., Yuan H., Tao X., Luo J. (2025). Developing High-Performance Anode-Free Lithium Batteries: Challenges, Strategies, and Opportunities. Adv. Funct. Mater..

[cit43] Tang Y., He J., Peng J., Yang J., Wu Z., Liu P., Zhou K., Hu S., Hu L., Wang X. (2024). Electrochemical Behavior of the Biomass Hard Carbon Derived from Waste Corncob as a Sodium-Ion Battery Anode. Energy Fuels.

[cit44] Yan M., Qin Y., Wang L., Song M., Han D., Jin Q., Zhao S., Zhao M., Li Z., Wang X., Meng L., Wang X. (2022). Recent Advances in Biomass-Derived Carbon Materials for Sodium-Ion Energy Storage Devices. Nanomaterials.

[cit45] Lv W., Wen F., Xiang J., Zhao J., Li L., Wang L., Liu Z., Tian Y. (2015). Peanut shell derived hard carbon as ultralong cycling anodes for lithium and sodium batteries. Electrochim. Acta.

[cit46] Zhang Z., Zhang A., Wang S., Sun J., Hou L., Yuan C. (2025). Biomass-derived hard carbon with tunable microstructures for sustainable and high-rate sodium-ion batteries. New J. Chem..

[cit47] Zhang Z., Li X., Dong P., Wu G., Xiao J., Zeng X., Zhang Y., Sun X. (2018). Honeycomb-like hard carbon derived from pine pollen as high-performance anode material for sodium-ion batteries. ACS Appl. Mater. Interfaces.

[cit48] Wang P., Zhu X., Wang Q., Xu X., Zhou X., Bao J. (2017). Kelp-derived hard carbons as advanced anode materials for sodium-ion batteries. J. Mater. Chem. A.

[cit49] Kim M., Fernando J. F. S., Li Z., Alowasheeir A., Ashok A., Xin R., Martin D., Nanjundan A. K., Golberg D. V., Yamauchi Y., Amiralian N., Li J. (2022). Ultra-stable sodium ion storage of biomass porous carbon derived from sugarcane. Chem. Eng. J..

[cit50] Lou F., Wang J., Wang X., Zhang M., Yuan J. (2025). Unraveling multi-level porous carbon negative electrode materials based on Rosa roxburghii pomace for high-performance sodium-ion batteries. RSC Adv..

[cit51] Wei C., Dang W., Li M., Ma X., Li M., Zhang Y. (2023). Hard-soft carbon nanocomposite prepared by pyrolyzing biomass and coal waste as sodium-ion batteries anode material. Mater. Lett..

[cit52] Wei H., Cheng H., Yao N., Li G., Du Z., Luo R., Zheng Z. (2023). Invasive alien plant biomass-derived hard carbon anode for sodium-ion batteries. Chemosphere.

[cit53] Chen S., Tang K., Song F., Liu Z., Zhang N., Lan S., Xie X., Wu Z. (2022). Porous hard carbon spheres derived from biomass for high-performance sodium/potassium-ion batteries. Nanotechnology.

