# Supplementary material for: An oxygen doped porous hard carbon derived from durian shell for high-performance sodium ion storage
Source: RSC Adv. 2026 Feb 16;16(10):9214–23. doi: 10.1039/d5ra09761d (PMC12908003; doi:10.1039/d5ra09761d)
Supplement: RA-016-D5RA09761D-s001 [file RA-016-D5RA09761D-s001.pdf]

Supplementary Information (SI) for RSC Advances.
This journal is © The Royal Society of Chemistry 2026

Supporting information

An oxygen doped Porous hard carbon derived from durian shell for high-performance sodium ion storage

Jianliang Guo,[†,‡] Zhihua Sun,[‡] Wenzheng Zhu,[‡] Lei Li,[‡] Song Han,[†,*] *Hongxun Yang,*[‡,*]

[†]*National Energy Group Science and Technology Research Institute Co., Ltd., Nanjing 210008, Jiangsu , China;* [‡]*School of Environmental & Chemical Engineering, Jiangsu University of Science and Technology, Zhenjiang 212003, Jiangsu, China*

*Corresponding author:

E-mail: yhongxun@126.com;

F-mail: 12068942@ceic.com

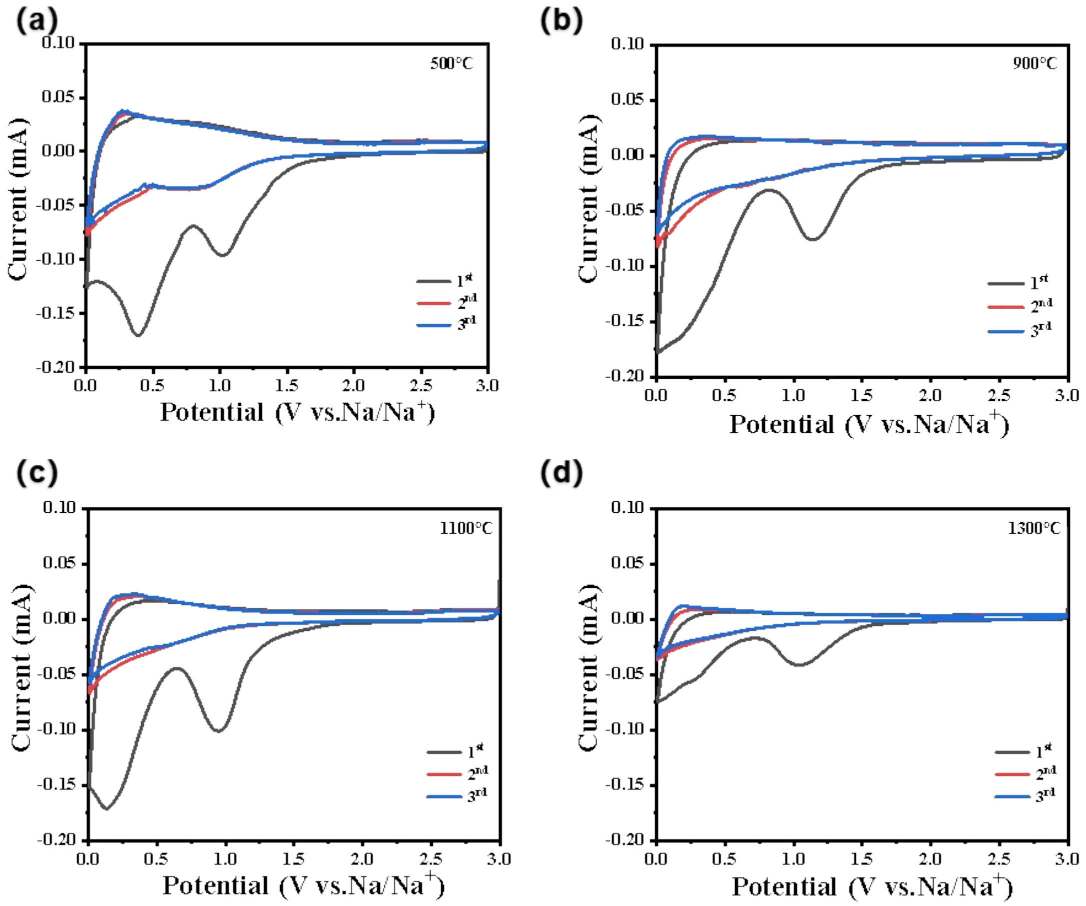

Fig. S1 (a-d) The CV curves of the first four electrodes of DSHC500, DSHC900, DSHC1100 and DSHC1300 at a scanning rate of 0.1 mV s^{-1} .

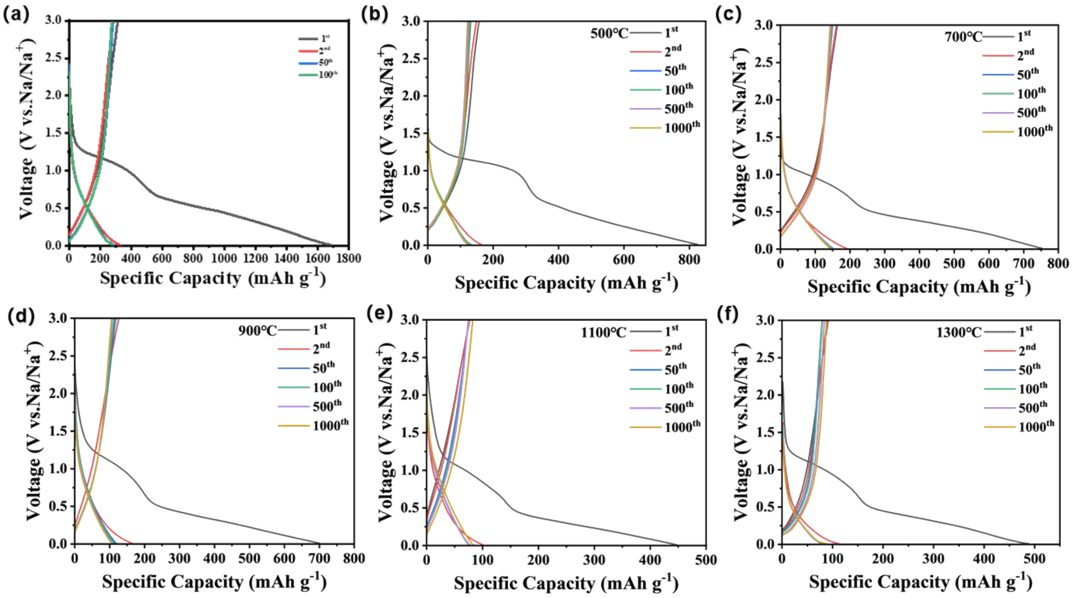

Fig. S2 (a) The 1st, 2nd, 50th and 100th charge-discharge curves of DSHC700 at 25 mA g^{-1}; (b-f) The 1st, 2nd, 50th, 100th, 500th and 1000th charge-discharge curves of DSHC500, DSHC700, DSHC900, DSHC1100 and DSHC1300 of different temperature at 500 mA g^{-1}.

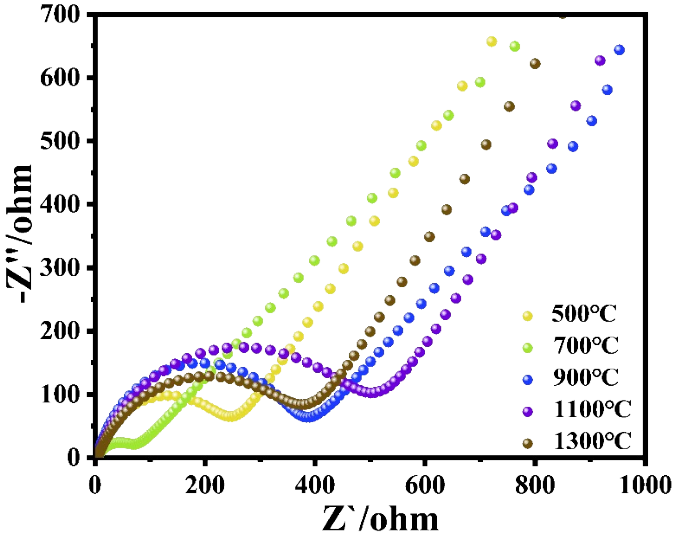

Fig. S3. The EIS spectra of DSHC-x after cycling.

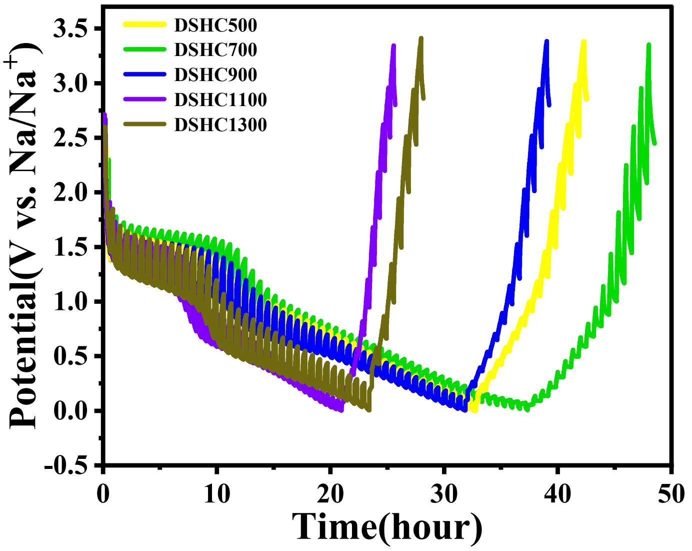

Fig. S4. GITT curves of DSHC-x.

Table S1. d_{002}, I_D/I_G, L_a of DSHC-x series.

	DSHC500	DSHC700	DSHC900	DSHC1100	DSHC1300
d_{002}(nm)	0.378	0.382	0.391	0.367	0.362
I_D/I_G	1.03	1.013	1.003	1.11	1.08
L_a(nm)	19.697	18.978	19.167	17.319	17.801

Table S2. Comparsion of the electrochemical performance of DSHC700 in this work and the previously reported hard carbon materials based anode materials

Samples	Initial coulumbic efficiency (ICE)	Reversible capacity (mAhg^{-1})	Current Density (mAg^{-1})	Cycle number	Ref.
DSHC700	18.5%	280.2	25	100	**This work**
CDHC-1300	80%	250.3	25	100	43
LPC-800	\	277.5	25	100	44
PSDHC-600	\	256.5	25	100	45
RPHC-1200	64%	224.3	25	100	46
CPP	59.8%	221.5	100	200	47
KHC-1300	64%	205	200	300	48
SC-800	78.2%	230	50	50	49
RRP-800-N	99.91%	223.7	31	100	50
HC-SC	92.3%	261	50	100	51
SAL	58.9%	245.3	50	100	52
SCL	88.1%	207.2	50	100	52
ECL	69.7%	227.1	50	100	52
CSHP2	44.1%	215	100	100	53

Reference:

[1] Y. Tang, J. He, J. Peng, J. Yang, Z. Wu, P. Liu, K. Zhou, S. Hu, L. Hu, X. Wang, Electrochemical Behavior of the Biomass Hard Carbon Derived from Waste Corncob as a Sodium-Ion Battery Anode, Energy Fuels, 38(2024) 7389-7398.

[2] M. Yan, Y. Qin, L. Wang, M. Song, D. Han, Q. Jin, S. Zhao, M. Zhao, Z. Li, X. Wang, L. Meng, X. Wang, Recent Advances in Biomass-Derived Carbon Materials for Sodium-Ion Energy Storage Devices, Nanomaterials, 12(2022) 936.

[3] W. Lv, F. Wen, J. Xiang, J. Zhao, L. Li, L. Wang, Z. Liu, Y. Tian, Peanut shell derived hard carbon as ultralong cycling anodes for lithium and sodium batteries, Electrochim. Acta,176(2015) 533-541.

[4] Z. Zhang, A. Zhang, S. Wang, J. Sun, L. Hou, C. Yuan, Biomass-derived hard carbon with tunable microstructures for sustainable and high-rate sodium-ion batteries, New. J. Chem. 49 (2025) 6277-6287.

[5] Z. Zhang, X. Li, P. Dong, G. Wu, J. Xiao, X. Zeng, Y. Zhang, X. Sun, Honeycomb-like hard carbon derived from pine pollen as high-performance anode material for sodium-ion batteries, ACS Appl. Mater. Interfaces, 10 (2018) 42796-42803.

[6] P. Wang, X. Zhu, Q. Wang, X. Xu, X. Zhou, J. Bao, Kelp-derived hard carbons as advanced anode materials for sodium-ion batteries, J. Mater. Chem. A, 5 (2017) 5761-5769.

[7] M. Kim, J. F. S. Fernando, Z. Li, A. Alowasheeir, A. Ashok, R. Xin, D. Martin, A. K. Nanjundan, D. V. Golberg, Y. Yamauchi, N. Amiralian, J. Li, Ultra-stable sodium ion storage of biomass porous carbon derived from sugarcane, Chem. Eng. J., 445 (2022) 136344.

[8] F. Lou, J. Wang, X. Wang, M. Zhang, J. Yuan, Unraveling multi-level porous carbon negative electrode materials based on Rosa roxburghii pomace for high-performance sodium-ion batteries, RSC Adv., 15 (2025) 20872-20880.

[9] C. Wei, W. Dang, M. Li, X. Ma, M. Li, Y. Zhang, Hard-soft carbon nanocomposite prepared by pyrolyzing biomass and coal waste as sodium-ion batteries anode material, Mater. Lett., 330 (2023) 133368.

[10] H. Wei, H. Cheng, N. Yao, G. Li, Z. Du, R. Luo, Z. Zheng, Invasive alien plant

biomass-derived hard carbon anode for sodium-ion batteries, Chemosphere, 343 (2023) 140220.
[11] S. Chen, K. Tang, F. Song, Z. Liu, N. Zhang, S. Lan, X. Xie, Z. Wu, Porous hard carbon spheres derived from biomass for high-performance sodium/potassium-ion batteries, *Nanotechnology*, 33 (2022) 055401.

Table S3. Impedance fitting values of DSHC-x electrodes before and after cycling.

	Before cycling		After cycling	
	R_s (Ω)	R_{ct} (Ω)	R_s (Ω)	R_{ct} (Ω)
DSHC500	2.19	647.2	1.33	266.3
DSHC700	2.55	436.8	0.92	93.2
DSHC900	2.16	230.6	1.01	200.2
DSHC1100	1.55	335.4	1.34	318.8
DSHC1300	4.54	316.3	1.38	277.2

Text S1

(1) Calculation formula for d_{002}

$$d_{002}=0.5\lambda/\sin\theta \quad (1)$$

Where λ is the K_α wavelength of Cu (λ = 0.15416 nm), θ is the diffraction angle.

Text S2

(2) Calculation formula for L_a

$$L_a(\mathrm{nm}) = \left(2.4 \times 10^{-10}\right) \times \lambda^4 \left(\frac{I_D}{I_G}\right)^{-1} \quad (2)$$

Where λ = 532 nm is the laser wavelength in nanometers.

Text S3

(3) Relationship between peak current i and scan rate v

$$I = a*v^b \quad (3)$$

$$\log i=\log a + b*\log v \quad (4)$$

Where a and b are adjustable empirical constants, the value of b can be obtained from the slope of log(i) and log(v). Generally speaking, a and b value close to 0.5 indicates that the diffusion process mainly controls the electrochemical reaction, while a and b value close to 1 represents an ideal capacitive process.

Text S4

(4) Contribution equation of pseudo-capacitance behavior to sodium cell

$$i\ (V) = k_1 v + k_2 v^{0.5} \quad (5)$$

$$i(V)/v^{0.5} = k_1 v^{0.5} + k_2 \quad (6)$$

Where i is the current, v is the sweep speed, k_1 and k_2 are constants under a certain voltage, $k_1 v$ and $k_2 v^{1/2}$ represent the current contributed by diffusion behavior and pseudocapacitance behavior respectively. At a specific sweep speed, the corresponding values ofk_1 and k_2 at a certain voltage can be obtained by fitting the linear relationship in formula (4), so as to calculate the current value contributed by the diffusion behavior and pseudocapacitance behavior.

Text S5

(5) According to Fick's second law, D_{Na^+} can be calculated using the following simplified equation.

$$D_{Na^+} = \frac{4}{\pi\tau}\left(\frac{m_B V_m}{M_B S}\right)^2\left(\frac{\Delta E_s}{\Delta E_\tau}\right)^2 \quad (7)$$

where m_B represents the weight of the active material on the electrode, V_m represents the molar volume of carbon, M_B represents the molecular mass of carbon, S represents the area of the HCRH-1200 electrode, τ represents the pulse time, and ΔE_s represents the voltage difference when the voltage reaches a steady state during a single GITT process.
